# New genes from non-coding sequence: the role of de novo protein-coding genes in eukaryotic evolutionary innovation

**DOI:** 10.1098/rstb.2014.0332

**Published:** 2015-09-26

**Authors:** Aoife McLysaght, Daniele Guerzoni

**Affiliations:** Smurfit Institute of Genetics, University of Dublin, Trinity College Dublin, Dublin 2, Republic of Ireland

**Keywords:** de novo genes, proto-genes, open reading frame, evolution

## Abstract

The origin of novel protein-coding genes de novo was once considered so improbable as to be impossible. In less than a decade, and especially in the last five years, this view has been overturned by extensive evidence from diverse eukaryotic lineages. There is now evidence that this mechanism has contributed a significant number of genes to genomes of organisms as diverse as *Saccharomyces*, *Drosophila*, *Plasmodium*, *Arabidopisis* and human. From simple beginnings, these genes have in some instances acquired complex structure, regulated expression and important functional roles. New genes are often thought of as dispensable late additions; however, some recent de novo genes in human can play a role in disease. Rather than an extremely rare occurrence, it is now evident that there is a relatively constant trickle of proto-genes released into the testing ground of natural selection. It is currently unknown whether de novo genes arise primarily through an ‘RNA-first’ or ‘ORF-first’ pathway. Either way, evolutionary tinkering with this pool of genetic potential may have been a significant player in the origins of lineage-specific traits and adaptations.

## 1. Introduction

A persistent and fundamental question in evolutionary genetics concerns the origin of genetic novelty [1–3]. Although it is possible for novel functions to arise within an existing gene [4], it is likely that there will be some degree of antagonism or adaptive conflict between the new and the old functions (e.g. [3,5]). By contrast, new loci are free of such constraints and constitute genetic novelty that may form the basis for lineage-specific adaptations and diversification [6–8].

The most radical form of genetic novelty comes from genes that originate de novo from non-genic DNA in that they are not similar to any pre-existing genes. Both protein-coding and RNA genes are important, but for the purposes of this perspective we will only consider the former.

Clearly, protein-coding genes must have arisen de novo from non-coding sequence in very early life evolution. However, it is likely that the processes of evolution once life was established were very different from those processes that established life [9]. Consequently, de novo origin was usually considered so improbable as to be impossible for more recent evolution [2,8]. Instead, gene duplication, fusion and fission of genes, exon shuffling and other ‘bricolage’ events were considered to be the only viable sources of novel protein-coding genes—all variations on a genetic theme [9]. Proteins were thought to be made from a small and finite ‘universe of exons' [10]. François Jacob articulated this best when he said ‘To create is to recombine’ [9]. However, in recent years, there has been a growing appreciation for the role of de novo gene origination.

## 2. Recent and ongoing de novo gene origination

Until quite recently, most known examples of novel peptide sequences were intimately related to a pre-existing gene, usually being an extension of coding sequence into an intron or UTR, or, more radically, translating an alternative reading frame of the mRNA in so-called ‘overprinting’ [8,11–15]. However, it has now become clear that de novo origin of protein-coding genes from non-coding DNA is a consistent feature of eukaryotic genomes, having been discovered in organisms as diverse as yeast, plants, flies, mammals, primates and even in recent human evolution (table 1).

**Table 1.** Recently originated de novo genes discovered in diverse eukaryotic lineages**.**

organisms	number of de novo genes	genes found in previous studies	notable examples and comments	references
*Drosophila*				
*D. melanogaster*	5	—	four are X-linked; all five have testis expression bias	[16]
*D. yakuba* and *D. erecta*	7 + 3	—		[17]
mainly *D. Yakuba*	11	—	seven are X-linked	[18]
*D. melanogaster* subgroup	1	—	*hydra*; testis expression	[19]
*D. melanogaster* subgroup	14	5	—	[20]
*D. melanogaster* group and *D. willistoni*	16	—		[21]
*D. melanogaster*	248 (106 fixed) proto-genes	—	discovered based on testis expression. Male-biased and underrepresented on X chromosome	[22]
mammals				
primates (*H. sapiens, P. troglodytes, M. mulatta)*	15	—	*PART1;* prostate carcinogenesis	[23]
hominoids	24	2	regulated RNA expression predates protein-coding potential. Transcription in cerebellum	[24]
hominids	1	—	*NCYM;* neuroblastoma pathogenesis	[25]
*H. sapiens*	3	—	*CLLU1;* upregulated in chronic lymphocytic leukaemia	[26]
*H. sapiens*	1	—	*FLJ33706* (*C20orf203*); expressed in brain; protein found in neurons.	[27]
*H. sapiens*	60	1		[28]
*H. sapiens*	1	—	*PBOV1;* mitigates cancer outcomes	[29]
*H. sapiens*	1		*ESRG;* essential for maintenance of pluripotency	[30]
*M. musculus*	1	—	*Poldi*; testis expression	[31]
*M. musculus* and *R. norvegicus*	69 + 6	—		[32]
plants				
*Oryza*	1	—	*OsDR10*; defence gene	[33]
*A. thaliana*	1	—	*QQS*; starch biosynthesis pathway	[34]
*A. thaliana and* Brassicaceae	25			[35]
*Plasmodium*				
*P. vivax*	13	—	5/13 have introns within the coding sequence	[36]
Yeast				
*S. cerevisiae*	1	—	*BSC4*; DNA repair, synthetic lethal	[37]
*S. cerevisiae*	1	—	*MDF1*; functional role in promoting vegetative growth	[38]
*S. cerevisiae*	1	—	*RDT1*; ORF is absent in some strains of *S. cerevisiae*	[39]
*S. cerevisiae*	∼1900 proto-genes	—		[40]

The evidence for de novo genes started to accumulate in the last decade. In 2006, Begun and colleagues presented evidence for de novo genes in *Drosophila* [16,17]. The first functional characterization of a gene known to be of recent de novo origin came in 2008 when Cai *et al*. [37] showed that *BSC4* in *Saccharomyces cerevisiae* has a role in DNA repair and is a synthetic lethal. Even in the absence of precise functional annotation, several de novo genes in flies and mammals have been shown to be under selection (e.g. [22,31]) a sure sign that they are contributing to fitness. Finally, the first population genetics study of de novo genes clearly demonstrated that de novo genes are continuously arising and many are still polymorphic [22].

Though de novo gene origination has gained widespread acceptance as a phenomenon in recent eukaryotic evolution [41], the extent of its impact remains to be discovered.

## 3. De novo genes in primates

There is perhaps a special interest in the discovery of de novo genes in the human genome and our close relatives. These genes are potentially involved in important lineage-specific adaptations. However, they are also unusual in having no homologues in model organisms, which is a major obstacle to understanding their functional contribution, if any.

Most of the genes inferred in primate genomes are annotated with reference to the human genome. This introduces a bias in gene annotation that is likely to overlook genes specific to non-human lineages, and over-infer orthologues of human genes. Thus, the identification of truly novel human genes is not trivial and can lead to many false positives and false negatives.

Most de novo genes identified in human and primates remain uncharacterized. However, several studies found that human or hominoid de novo genes are most abundantly expressed in brain tissue, which at least hints at a role of these new genes in brain evolution [24,27,28].

Ever since the first discovery of human-specific de novo genes, there have been suggestive but weak links with disease [23,26,27]. Recently, *NYCM*, a de novo gene present exclusively in human and chimpanzee genomes, was shown to be involved in the pathogenesis of human neuroblastoma through interaction with the oncogene *MYCN* [25]. Additionally, knockdown of a transcript containing a human-specific de novo open reading frame (ORF) that originated within an endogenous retrovirus revealed that at least the transcript is essential for the maintenance of pluripotency [30]. These provide the first experimental evidence of the functional importance of de novo genes in our own species.

We carried out an independent analysis to identify protein-coding genes in human and Homininae. Our criteria were purposely very strict to avoid inclusion of ambiguous cases such as those hinting at protein elongations or those cases where recent independent gene losses could not be excluded. We found a total of 35 de novo candidates: 16 human-specific, 5 human + chimp-specific and 14 Homininae-specific (D Guerzoni and A McLysaght, manuscript in preparation). These counts are roughly proportional to branch lengths and thus support the inference of a relatively constant rate of de novo gene acquisition in this lineage.

## 4. Identification of de novo genes

The numbers of genes detected vary quite widely from study to study with very little overlap (table 1). For example, the first report of human-specific de novo genes predicted around 18 such genes should exist [26], whereas a more recent paper identified 60 [28]. These differences are due to the volatility of the annotation of lineage-specific genes but also due to differences in the search strategies adopted in different studies [42]. This shift in methods of detection reflects the growing acceptance of the possibility of de novo gene origination: whereas the first papers in the field were cautious and conservative in terms of reporting de novo genes, more recent papers assume de novo genes exist and employ less conservative search strategies as they seek to assess their evolutionary impact. However, it is still the case that careful curation of lists of de novo genes is required if we are to gain a proper understanding of the extent of their specific contribution to recent evolution and how they acquired functionality.

De novo genes are usually defined as protein-coding genes that have evolved from scratch from previously non-coding DNA. There are significant challenges surrounding the accurate detection of de novo genes. Identification of de novo genes generally starts with a sequence similarity search in the genomes of closely related organisms. The failure to detect a homologous gene in a sister lineage is the first piece of evidence in support of the de novo origins of the gene of interest. We are interested in detecting cases where the gene is absent because it evolved after the lineage divergence. However, we must also contemplate and eliminate the alternative possibilities that the absence is due to recent gene loss in the sister lineage, or that the absence is false and is in fact an annotation omission or genome sequencing gap. For these reasons, the most rigorous (and conservative) methods to detect de novo genes require positive evidence of the absence of the gene in the other lineages (such as the identification of orthologous but non-coding sequence), thus permitting inference of absence in the ancestral sequence [26,42]. Ideally, these studies should include transcriptome data analysis to accompany DNA sequence analysis to minimize the under-discovery of genes.

It is possible that some of the more conservative search criteria introduce bias into the results. For example, the prediction of intron–exon boundaries in the absence of supporting evidence is problematic. There is therefore a real challenge to determine whether a potential early stop codon is in frame, thereby eliminating the ORF from consideration, or if it is in an intron of a valid candidate gene. Many of the detected de novo genes have only a single coding exon, which may be a genuine reflection of their simple structure, or an artefact introduced by the search strategy, or a mixture of both. (The virtual absence of introns in *S. cerevisiae* should ensure an avoidance of this particular problem in analyses of that genome.)

Similarly, many de novo genes have been discovered close to or overlapping older genes. This may reflect a reuse of pre-existing regulatory sequences [26,43] or conservative search criteria that require detection of orthologous but non-coding DNA in an outgroup lineage. The sequence conservation that enables detection of orthology is more likely if there is functional constraint on an overlapping sequence. This problem could be avoided by only considering the non-overlapping region of the novel ORF for the purposes of the sequence similarity search.

By contrast, liberal search criteria naturally carry the risk of a high false positive rate, and some do not make the distinction between extension of a gene into previously non-coding sequence and entirely de novo origination [42]. Eukaryotic genomes may carry a large number of ORFs that are not annotated as genes, many of which might naively be considered as candidate de novo genes. For example, the *S. cerevisiae* genome contains about 261 000 unannotated ORFs of at least three codons long [40]. We searched the human genome for ORFs and found over 13.5 million ORFs of at least 33 codons long, compared to over 47 000 of the same length threshold in yeast (including annotated genes). This increase is roughly proportionate to the larger genome size in human but is extremely disproportionate to the number of annotated genes. This suggests that the problem of false positives may be more acute in the human genome and other large genomes.

Recently, Zhao *et al*. [22] adopted a different strategy to search for de novo genes. They used RNA-seq in *Drosophila* to characterize species-specific transcripts, and examined these for evidence of natural selection and the presence of ORFs. Over half of the candidate de novo genes discovered in this study are not fixed and many of them will probably be lost from the population. Even so, they uncovered a larger number of candidate de novo genes than any earlier study, which is even more remarkable given that they only examined one tissue. This suggests that there remains a large number of undiscovered potential de novo genes.

## 5. Steps in the de novo origin of genes

In order for non-coding DNA to begin to function as a protein-coding gene, an ORF must originate, the DNA must be transcribed and the mRNA translated, and the protein should ultimately become integrated into the cellular processes. Though it is tempting to think of this as a stepwise, directional process, the evidence from yeast and from flies is that there is a reversible evolutionary continuum from non-gene to gene [40,44,45]. Those sequences in the grey-zone between non-genes and genes have been termed ‘proto-genes' by Carvunis *et al*. [40].

The earliest discoveries of de novo genes, though from very different lineages, all had one thing in common—the identified genes were short and simple. This observation led to the suggestion that the emergence of de novo genes should be a gradual process, and that these examples were neonates [43]. In keeping with this, proto-genes gradually acquire traits characteristic of genes such as longer coding length, higher expression, *cis*-regulatory sequences, codon usage bias and purifying selection [40]. Similarly, the encoded proteins get progressively integrated into cellular processes [45,46]. Furthermore, young de novo genes that are polymorphic in *Drosophila melanogaster* and ‘caught in the act’ of originating were significantly shorter and simpler than annotated genes [22].

In order to be considered a candidate de novo protein-coding gene, these sequences must both contain an ORF and be expressed; however, there is no reason to think that these must arise in a particular order [47].

**Figure 1.**
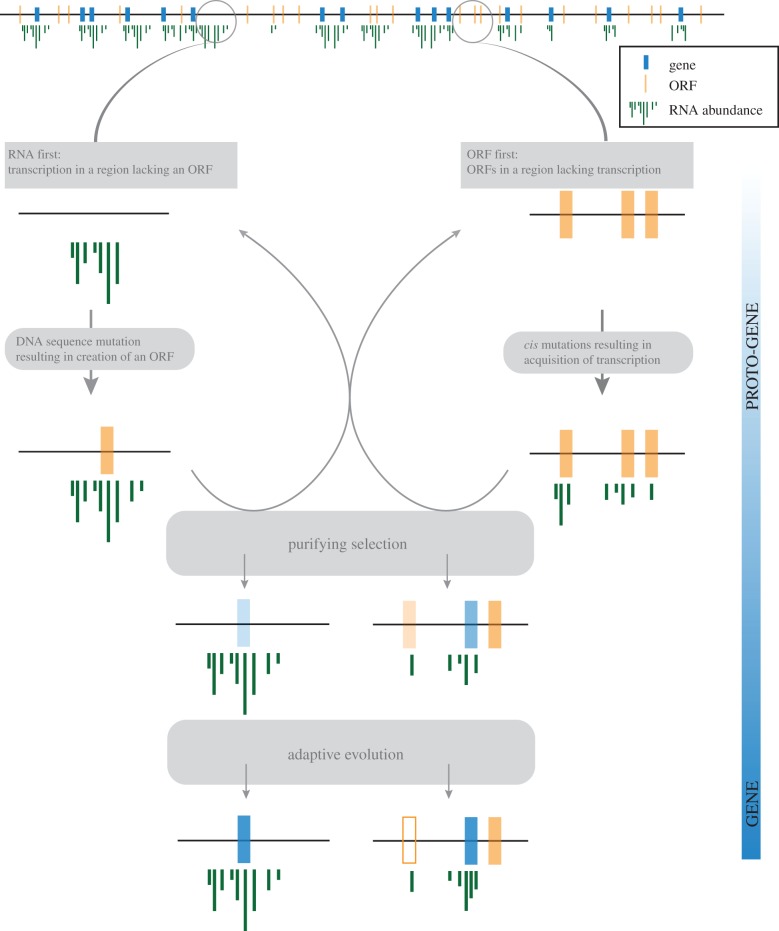
Dynamic and reversible de novo evolution of genes. A huge amount of potential within large eukaryotic genomes exists in the form of expressed non-coding regions (left) and non-expressed ORFs (right). DNA sequence mutations can create an ORF in already expressed regions, or give rise to *cis*-regulatory signals in regions already containing an ORF. Purifying selection can act as a filter to remove the most deleterious cases, either by abolition of expression or disruption of the ORF. Remaining proto-genes may become true genes through the action of positive selection and/or drift. Drift may operate at any point in the process and is omitted for visual clarity.

An RNA-first model (figure 1, left) describes a transcribed region of genome which acquires an ORF through DNA mutations [24]. This scenario is supported by multiple observations of de novo genes where the orthologous region in a sister lineage is transcribed but there is no ORF, suggesting that the ancestral sequence was transcribed prior to the emergence of the ORF [20,37]. There is also strong evidence that RNAs such as lncRNAs can provide a ready supply of new peptides [24,47,48]. The discovery that five out of 13 de novo genes in *Plasmodium vivax* have introns within the coding sequence, even given the unusual evolutionary constraints on introns in that genome, led to the suggestion that the complex intron–exon structure predated the coding capacity of these loci, probably as features of an RNA gene [36]. In these cases, only the protein-coding capacity can be said to be de novo.

Alternatively, given the large number of ORFs per genome it is easy to imagine how an existing ORF might eventually become expressed (figure 1, right). Novel DNA sequence changes in regions *cis* to ORFs can induce expression [2]. Not only ORFs but other gene features may be cryptic in the genome. In the case of the mouse de novo gene *Poldi*, there is evidence that some of the complex gene structures involved in regulation and splicing predate the expression of the locus [31].

The transcription-first model appears to be more popular, having been the first to accumulate evidence. However, the first study of the population genetics of de novo genes found evidence for pre-existing ORFs becoming expressed [22]. Zhao *et al.* identified loci that harbour ORFs in all *D. melanogaster* individuals and also in sister lineages but where transcription was only discovered in a subset of individuals. The expression polymorphism was linked to *cis-*sequence variation [22]. These results show a clear mechanism for previously cryptic ORFs in the genome to become expressed.

## 6. Fixation of de novo genes

The fixation of a de novo gene is expected to have important differences from the fixation of genes formed by re-use of existing genes either in part or in their entirety [21,49]. In the case of gene duplication, the new gene is redundant and in most cases carries no immediate selective advantage or disadvantage, and although it is functional, there is no novelty involved. As such, initial fixation will often be largely dictated by passive processes rather than selection [50]. Genes generated by fusion, fission or recombination will create some immediate novelty, but the component parts are likely to retain the functionality of the protein domains that they contain, albeit in a novel context, some of which may confer an immediate selective advantage or disadvantage.

The potential protein of a novel ORF can be considered an arbitrary sequence, as opposed to one that has been refined by natural selection. It has been shown that an arbitrary sequence can contain selectable variation, at least in some circumstances [51]. If not expressed, neither the favourable nor the unfavourable ORFs in the genome will have the opportunity to be improved or removed by selection. If an arbitrary ORF abruptly became highly expressed, it is improbable that it would have a positive effect, and perhaps more likely that it would be deleterious [16], especially if it is long. However, at low levels of expression such as is typical for proto-genes [40], these regions could become exposed to selection to remove deleterious proto-genes before they become established [39,52]. Thus, the pool of proto-genes is enriched for those with a more plausible chance of becoming a gene [39]. Such a scenario enhances the probability that a proto-gene can successfully transition to a gene.

Genetic drift has played a large role in the evolution of complex genomes. Eukaryotic genomes have accumulated many initially sub-optimal features which, though they originate passively, do ultimately confer adaptive potential in the form of genetic raw material [53]. Population size is considered to have been a determining factor in this because in small populations the distribution of fitness effects is altered so that a larger proportion of variants is effectively neutral [53,54]. Similarly, it will be interesting to explore the impact of population size on the rates of de novo gene origination.

There is evidence for de novo gene origination by both ‘RNA-first’ and ‘ORF-first’ routes. At present, there are not sufficient data to determine whether one of these is a more productive source of new genes. A certain fraction of the arbitrary peptides generated in this way will be deleterious [9,55]. We may thus imagine two scenarios: one where an arbitrary ORF appears in a locus of significant transcription (‘RNA first’) and one where a cryptic, arbitrary ORF experiences some low, perhaps sporadic, transcription (‘ORF first’).

In both scenarios, transcription is required to expose the genomic variation to natural selection. In the ‘RNA-first’ scenario, the transcript regulation and processing might be refined and stabilized by natural selection prior to the emergence of the ORF. The initial translation of the ORF might be no more than noise, but such noise could permit the removal of strongly deleterious ORFs by natural selection [39]. lncRNAs might be particularly suited to act as the foundations for de novo genes, because they have only limited sequence constraints [56] and so limited adaptive conflict with the evolution of an ORF.

In the ‘ORF-first’ scenario, the transcript and the ORF potentially become exposed to natural selection simultaneously. Under this model, the ORF is already fixed and transcription may either be initially just noise [57], or may be induced by *cis* mutations [22] and so be initially stable but polymorphic. Ribosomes can associate with these transcripts [39,40,48,58,59] and so a similar opportunity for purifying selection exists.

Both RNA-first and ORF-first provide plausible routes for the evolution of new genes. In either case, the final steps will be determined by a combination of drift and selection. Whether one route is favoured over the other will depend on the size of the ‘mutation space’ that can generate an ORF from non-coding sequence (RNA first), or that can induce expression in a silent region of the genome (ORF first). Effective population size (*N*_e_) could also have an impact on which route is favoured. In larger populations, selection is more effective and drift is weak. In such circumstances, the RNA-first route might be more plausible because the initial steps can involve fixation of a functional RNA gene through positive selection. By contrast, at small *N*_e_ the genome is more likely to be large and noisy, and this could increase the opportunities for the ORF-first route.

## 7. Functional contribution of de novo genes

In the *Descent of Man*, Darwin draws a distinction between a difference of ‘degree’ and a difference of ‘kind’. In the same way, we can consider whether apparently lineage-specific traits are the result of genes that are different by degrees (diverged form of a gene present in the common ancestor) or of a different kind (de novo genes). Phylostratigraphic studies of eukaryotic genomes have pointed to several evolutionary periods that have disproportionately experienced a high rate of emergence of new genes [7]. These periods are associated with major species radiations and thus support the notion that new genes are integral to evolutionary innovation.

A large part of the interest in de novo genes is to do with understanding their potential to evolve novel functions in a relatively short time-frame. There are a few examples of de novo genes with well-characterized functionality. The human-specific de novo gene *FLJ33706* was discovered to be most highly expressed in brain tissue and was furthermore found to have elevated levels in Alzheimer's disease brain tissue, and a single-nucleotide polymorphism within the gene has been linked to addiction disorders [27]. Knockdown experiments demonstrated that the novel, human-specific gene *ESRG* is required for the maintenance of pluripotency in human naive stem cells [30]. It is difficult to definitively show that it is the peptide rather than the RNA that is functional, but these experimental results are encouraging.

*MDF1* is a de novo gene which is only found in *S. cerevisiae*. Li *et al.* [38] conducted several careful experiments to demonstrate that this very new gene has a function in suppressing sexual reproduction by binding MAT*α*2 in rich medium and thus promoting vegetative growth. More recently, it was shown that the link between nutrient availability and mating is mediated by *MDF1* through its function in two distinct pathways [60]. Thus, this novel gene has not only acquired functionality quite rapidly but has integrated into two central cellular processes.

The essentiality of de novo genes in *Drosophila* is currently less clear, because although one paper reported that out of 16 de novo genes examined three were essential for viability [21], it has subsequently been shown that the Vienna RNAi lines used in this and other papers may be compromised [61]. Thus, it remains to be seen whether or not these particular results are valid.

One important question concerns how a newly evolved gene can become essential. It is an apparent paradox because clearly the organism previously survived in the absence of that gene. It could be that coevolution of a de novo gene with an older gene interaction partner could lead to such essentiality [21]. It is also possible that the new gene might have provided an alternative function in the cell that resulted in relaxed constraint on some functions of other genes or pathways which were subsequently lost. Whereas duplicated genes may become essential by passive processes such as subfunctionalization, de novo genes can only become essential through neofunctionalization [21], a process which is expected to involve positive selection.

## 8. Open questions in de novo gene evolution

The study of de novo genes is a new field, and there is much that remains to be discovered. This is an exciting area of research because it offers a rare opportunity to witness the evolution of promoters, gene structure and protein function [45,62,63].

One interesting question concerns the biological processes where de novo genes become integrated. If there are trends or biases in where de novo genes become functional it could point towards some processes being more dynamic and open to integrating new genes.

In general, new genes have been shown to be biased towards male-specific expression or function, specifically in testis [2]. Haldane's rule (the observation that in cases of hybrid sterility it is usually the heterogametic sex that is sterile) is consistent with a model where genes involved in reproduction have a faster rate of evolution in the heterogametic sex [62]. Interestingly, several of the reported de novo genes have inferred male reproductive roles or expression bias [16–19,22,31,44].

It is also interesting to consider how the genome organization itself might influence the de novo origin of genes. De novo genes have been observed in the vicinity of other genes, leading to the suggestion that they might exploit the existing regulatory sequences of their neighbours [26,43]. In yeast, ORFs of different age classes frequently overlap each other, usually on the opposite strand [40]. One possible mechanism for pre-existing genes to influence de novo gene origin could be through a promoter becoming bidirectional [64]. Conversely, it has also been shown that de novo regulatory sequences can be associated with the emergence of a gene [22]. It is not yet known how important existing genes are as indirect ‘drivers' of the evolution of de novo genes.

Some regions of the genome have a particularly permissive expression environment which might facilitate the graduation of ORFs to proto-genes. One of the first human de novo genes discovered, *CLLU1* [26], is located in a region of high transcription [65]. The *Drosophila* X chromosome is hypertranscribed in males and early reports of de novo genes found an X chromosome bias [16,18]. This pattern, however, is not universal [44]. Other genomic features may facilitate the emergence of de novo genes. Transposable elements have been linked to the origin of the *hydra* gene in *Drosophila* [19] as well as some primate orphan genes [23]. In yeast, proto-genes are frequently located in sub-telomeric regions [40]. Some features of endogenous retroviruses may provide promoters and RNA processing signals [30].

We can consider the impact that this process has had on genome evolution. The aspect we have focused on so far is the origination of new genes. However, another potential impact is that there could be purifying selection on the presence of ORFs in transcribed loci, or equally on the transcription of ORF-containing loci. It would be interesting to test the interplay between the large number of ORFs present in our genome and the extensive transcription that has been experimentally observed. For example, if ORFs are rare in transcribed regions of the genome that would suggest the action of purifying selection.

Generally intractable by comparative genomics analysis, ribosome occupancy experiments have been powerful in the identification of small peptides. Recently, small polypeptides originating from short ORFs (as opposed to processed from a larger protein) have gained recognition as relevant and potentially numerous components of genomes [66,67]. The evolution of short ORFs de novo seems to be particularly plausible. It would not be surprising to discover a high turnover of generation and loss of novel short-peptide-encoding ORFs. With the cost of expression virtually nil, these have a reasonable chance of escaping the bottleneck of origination and becoming functional.

## 9. Concluding remarks

The discovery of de novo genes is more than simply a discovery of a set of genes in eukaryotic genomes, it is the discovery of the viability of this process that can release genomic variation for testing through the filter of natural selection. Given the large number of ORFs in eukaryotic genomes and the growing understanding of the importance of short peptides, it will be interesting to discover whether the underlying dynamics enable this pool of cryptic ORFs to have a significant evolutionary impact.

De novo genes are not only important for their functional and biological contribution to the lineages in which they originate; they are also very informative in terms of our growing understanding of the evolution the genome and of new gene functions. Evolution continues to tinker.
